# Crimes by people with schizophrenia in Korea: comparison with the general population

**DOI:** 10.1186/s12888-019-2355-5

**Published:** 2019-11-29

**Authors:** Agnus M. Kim

**Affiliations:** Department of Health Policy and Management, Seoul National University College of Medicine, 103 Daehak-ro, Jongno-gu, Seoul, 03080 South Korea

**Keywords:** Schizophrenia, Crime, Violence, Murder

## Abstract

**Background:**

This study was performed to describe the prevalence of crimes committed by persons with schizophrenia using population-based data and to compare the crime prevalence of persons with schizophrenia and the general population.

**Methods:**

The number of crimes was obtained from the Korean National Policy Agency (KNPA) crime statistics (2012–2016), which provide the number of crimes in terms of the criminal’s mental status and mental health conditions. For the number of persons with schizophrenia, estimates were used which had been calculated from the inpatient and outpatient claims from the National Health Insurance Service. The crime prevalence in persons with schizophrenia was calculated according to the types of crimes, and a comparison with the general population was conducted.

**Results:**

The overall crime prevalence of persons with schizophrenia was 72.7 to 90.3 per 10,000 from 2012 through 2016, which was about one fifth that of the general population. While the crime rates of the persons with schizophrenia were lower than the general population in most types of crimes including violence, intellectual crimes, and theft, the prevalence of murder, arson, and drug-related crimes in persons with schizophrenia was about five times, six times, and two times that of the general population respectively.

**Conclusion:**

The higher prevalence of serious offences among persons with schizophrenia suggests the need for closer and more appropriate care for the population, which would be achieved through effective continuity of institutional and community care.

## Background

The prevalence of crime in people with schizophrenia compared to the general population is an important issue both in psychiatry and law. It can be a measure that indicates the characteristics of the disease in terms of violence and impulsivity [1], and this tendency of crime with the disease, as reflected in the statistics, can be referred to in court for assessing the culpability of those with schizophrenia. In terms of mental health policy, the prevalence of crime in people with schizophrenia has serious implications in that schizophrenia is a major cause of psychiatric admissions [2–5] and especially that it is the largest cause of long-term hospitalizations [6, 7].

In the past half century, psychiatric care in many Western countries experienced a transition from institutionalization to community care, and this process of deinstitutionalization resulted in the dominance of community based care [8]. While this change was accepted as desirable in view of patient rights and the prevention of long-term hospitalization, concerns have been raised about the adaptation and integration of the patients [9], and the prevalence of crime in psychiatric patients have been investigated to address those concerns [10]. This issue is especially relevant in Korea which faces a challenge with the transition from inpatient-oriented psychiatric care.

Psychiatric care in Korea has long been highly dependent on inpatient care with high numbers of psychiatric beds and long lengths of stay [11]. This over-dependence on institutionalization in psychiatric care was related to a high rate of involuntary admission, which was made possible by the law [11]. As a result of increasing public awareness of involuntary admission and the rights of psychiatric patients, the Korean government revised the related law in 2016, altering the requirements for involuntary admission and lengthening of stays. However, this revision came under criticism in that it did not consider the possible consequences of returning psychiatric patients to society without due preparation.

Concern for the crimes by people with schizophrenia is at the core of this debate. Patients with schizophrenia account for about half of all psychiatric admissions and two thirds of long-term psychiatric admissions in Korea [2]. The inpatient expenditures for schizophrenia accounted for about 22% of the total psychiatric inpatient expenditures [12–14] with the average length of stay at 166 days in 2016 [15]. Most of all, there has been an ingrained prejudice about schizophrenia, which eventually led to the recent change of its Korean term to a more neutral one [12]. Moreover, frequent media coverage of the crimes committed by patients with schizophrenia has added to the concerns.

However, these individual crimes by schizophrenic patients should not be confused with their prevalence. Accurate assessment of the crimes by patients with schizophrenia in comparison with the general population would be a primary step toward addressing this concern. Although higher crime prevalence among people with schizophrenia than the general population has been documented in a number of studies [10, 13–15], a considerable discrepancy in the study results suggests that it is hard to arrive at a definite conclusion [13, 16]. This study was performed to describe the prevalence of crimes committed by persons with schizophrenia using population based data and to compare the crime prevalence between people with schizophrenia and the general population.

## Methods

### Data

The number of crimes was obtained from the Korean National Policy Agency (KNPA) crime statistics (2012–2016). The KNPA statistics are based on the electronic crime records obtained from all of the police offices in Korea in the course of police investigations [17]. It is composed of three kinds of statistics: Crime Occurrence Statistics, Crime Arrest Statistics, and Suspect Statistics. In this study, the Suspect Statistics were used which provide the most comprehensive information and are considered the basis of crime statistics in Korea [18]. The crimes are classified into 15 types, and among those, major crimes such as felonies, violence, and intellectual crimes have subdivisions. For example, felony has sub-types such as murder, robbery, rape, and arson, and intellectual crimes include fraud, embezzlement, and other financially motivated non-violent crimes [19]. The KNPA statistics provide the number of crimes in terms of the criminal’s mental status, which includes normal, mental disorder (schizophrenia), mental weakness (mental retardation), other mental disorders (bipolar disorder or personality disorder), drunkenness, and menstruation-related [20]. The crimes in the sub-category of mental disorder refer to those committed by persons with schizophrenia [21].

The population number was acquired from the Korean resident population of the corresponding year [22], and for the number of persons with schizophrenia, the estimate presented by the Health Insurance Review and Assessment was used [23]. The figures were calculated based on the inpatient and outpatient claims from the National Health Insurance Service, which covered the entire population in Korea [23]. The diagnosis codes for schizophrenia were derived from the definition in the Health Care Quality Indicators of the OECD [24], and the claims for which the principal diagnosis was schizophrenia were used to estimate the number of persons with schizophrenia.

### Prevalence of crime

The prevalence of crime by persons with schizophrenia and the general population were calculated per 10,000 persons of all ages. The numbers of crimes are classified by the mental status of the persons who committed them: normal, mental disorder, mental weakness, other mental disorder, drunkenness, and menstruation-related. As “mental disorder” is defined as having schizophrenia at the time of the crime by the KNPA [20], the number of crimes by those with “mental disorder” was used as the numerator and the estimated number of persons with schizophrenia as the denominator. In the case of the general population, the crime prevalence was calculated as the sum of the number of crimes committed by those classified as normal, mental weakness, other mental disorder, drunkenness, and menstruation-related divided by the total population except the estimated number of persons with schizophrenia. 

### Statistical analysis

The prevalence of crime in persons with schizophrenia and the general population were calculated by the types of crimes. In the cases of felony and violence, the prevalence was subdivided according to their sub-types. The proportion of crimes committed by persons with schizophrenia was compared with that of the total population from 2012 through 2016.

## Results

The numbers of persons with schizophrenia estimated from the National Health Insurance and Medical Aid claims were 254,586 in 2012 and 282,233 in 2016, and the prevalence of schizophrenia increased from 0.5 to 0.6 during the same period (Table 1). Table 2 presents the number of crimes committed by people with schizophrenia and the general population. The proportion of overall crimes committed by persons with schizophrenia was about 0.1%, which is about one fifth of the estimated prevalence of schizophrenia. However, in felonies, especially in murder, attempted murder and arson, the proportion of those with schizophrenia was about 0.7, 4.1, 2.9, and 3.7% respectively as of 2016. In addition, the proportion was high at 3.4% in drug-related crimes. However, in other types of crimes, the proportion was generally below the estimated proportion of schizophrenia.

**Table 1:** Number of persons with schizophrenia and the prevalence of schizophrenia in Korea (2012–2016)

	2012	2013	2014	2015	2016
N. of persons with schizophrenia	254,586	255,488	258,800	268,438	282,233
N. of population (total)	50,345,325	50,558,952	50,763,158	50,947,707	51,112,972
Prevalence of schizophrenia	0.5	0.5	0.5	0.5	0.6

**Table 2:** Number of crimes by persons with schizophrenia and the general population

Types of Crime	People with schizophrenia					General population				
	2012	2013	2014	2015	2016	2012	2013	2014	2015	2016
Overall	1850	1714	1879	2051	2549	1,718,517	1,735,444	1,706,170	1,764,410	1,839,318
Felonies^a^	132	160	174	173	188	23,096	24,553	24,199	24,915	26,098
Murder^a^	12	11	18	14	14	400	334	381	327	330
Attempted murder^a^	17	21	9	15	17	559	566	537	531	563
Robbery^a^	13	11	13	12	21	3239	2531	2003	1933	1633
Rape	60	77	87	76	89	17,596	19,870	19,953	20,753	22,338
Arson	30	40	47	56	47	1302	1252	1325	1371	1234
Violence	705	728	729	796	983	381,036	350,686	337,942	349,644	355,976
Theft	299	289	357	350	458	101,547	99,488	92,260	99,485	102,024
Intellectual crimes	115	81	83	102	107	245,994	267,498	253,012	259,734	253,278
Public order crimes	33	30	33	49	57	49,259	46,497	40,005	39,811	44,350
Drug-related crimes^a^	46	39	69	94	150	4560	4867	5022	6559	7923
Other crimes	520	387	434	487	606	913,025	941,855	953,730	984,262	1,049,669

^a^ refers to the crimes whose rates in people with schizophrenia were higher than in the general population

The prevalence of crime in persons with schizophrenia and the general population are presented in Table 3. The overall crime prevalence of persons with schizophrenia was 72.7 to 90.3 from 2012 through 2016, which was about one fifth that of the general population in those years. The increase in the overall crime prevalence between 2012 and 2016 was higher in persons with schizophrenia. While the crime prevalence in persons with schizophrenia was lower than the general population in most types of crimes including violence, intellectual crimes, and theft, the prevalence of murder, arson and drug-related crimes in persons with schizophrenia was about five times, six times, and two times that of the general population respectively.

**Table 3:** Prevalence of crime in persons with schizophrenia and the general population

Types of Crime	People with schizophrenia					General population				
	2012	2013	2014	2015	2016	2012	2013	2014	2015	2016
Overall	72.7	67.1	72.6	76.4	90.3	343.1	345.0	337.8	348.2	361.9
Felonies^a^	5.2	6.3	6.7	6.4	6.7	4.6	4.9	4.8	4.9	5.1
Murder^a^	0.5	0.4	0.7	0.5	0.5	0.1	0.1	0.1	0.1	0.1
Attempted murder^a^	0.7	0.8	0.3	0.6	0.6	0.1	0.1	0.1	0.1	0.1
Robbery^a^	0.5	0.4	0.5	0.4	0.7	0.6	0.5	0.4	0.4	0.3
Rape	2.4	3.0	3.4	2.8	3.2	3.5	4.0	4.0	4.1	4.4
Arson	1.2	1.6	1.8	2.1	1.7	0.3	0.2	0.3	0.3	0.2
Violence	27.7	28.5	28.2	29.7	34.8	76.1	69.7	66.9	69.0	70.0
Theft	11.7	11.3	13.8	13.0	16.2	20.3	19.8	18.3	19.6	20.1
Intellectual crimes	4.5	3.2	3.2	3.8	3.8	49.1	53.2	50.1	51.3	49.8
Public order crimes	1.3	1.2	1.3	1.8	2.0	9.8	9.2	7.9	7.9	8.7
Drug-related crimes^a^	1.8	1.5	2.7	3.5	5.3	0.9	1.0	1.0	1.3	1.6
Other crimes	20.4	15.1	16.8	18.1	21.5	162.2	166.4	167.4	170.5	182.3

^a^ refers to the crimes whose rates in people with schizophrenia were higher than in the general population

## Discussion

This study compared the prevalence of crime in persons with schizophrenia and the general population. While the overall crime prevalence was lower in persons with schizophrenia, they showed a markedly higher prevalence in murder, arson, and drug-related crimes. These results are consistent with the results of prior studies performed in Korea [15] and in the US and some European countries [10, 13, 14].

Before discussing our results, the characteristics of the data used and the process of our analysis need to be examined. Concerning the measurement of the prevalence of crime in persons with schizophrenia, the denominator, the number of persons with schizophrenia, was assessed with the claims database. Its point prevalence was estimated to be 0.5 to 0.6, which is close to and slightly higher than those reported in prior studies worldwide [25]. This signifies less room for over- or under-estimation of the crime prevalence due to under- or over-estimation of the number of patients with schizophrenia.

The number of crimes by people with schizophrenia, the numerator, was obtained from the records acquired during the course of police investigations. The diagnosis of the suspect is based on the statements of the suspect or his/her family. As this statement does not always come with the documents which can support it, the final assignment depends on the police officer who performed the investigation. Therefore, though having gone through the review by the police officer, the information provided by the suspect or his/her family can be susceptible to malingering.

However, allowing for the possibility of some over-estimation due to malingering, the record of the KNPA can still serve as a reliable estimate of the number of crimes by persons with schizophrenia. First, it is the official statistics of the Korean government which provide the most comprehensive crime record in Korea and undergo regular quality control. In addition, the specified types of mental status of the suspects in the statistics, such as other mental disorder or drunkenness, make malingered schizophrenia or misclassification less likely. Second, the number of court decisions, where the defendant’s having schizophrenia was acknowledged to affect the murder, was close to the number in our analysis [26]. According to a study by Choi et al., which analyzed the court decisions in Korea from 2014 to 2016 Nov., schizophrenia was referred to as a factor that affected the defendant’s committing a murder in a total of 37 cases. Given that the number of murders by persons with schizophrenia during the period which is one year before 2014 and 2016 Nov. is estimated to be 42 from the KNPA statistics, in about 90% of the murder cases where the suspect claimed to have schizophrenia, the defendant’s having schizophrenia and its influence on the murder were acknowledged in the court decision. Considering that the defendant’s schizophrenia is referred to in the court judgement only when it was judged to have influenced the occurrence of the crime and that there is often disagreement between the opinion of the judge and psychiatrist, the defendants in the remaining 10% cannot be considered not to have schizophrenia. It is more likely that the effect of the disease on the crime was not acknowledged by the judge than that the defendant did not have the disease. Above arguments suggest that, despite the possibility of malingering, the statistics presented in our study are reliable [27].

Even though the tentative “higher propensity of serious offense” is close to the realities, whether the higher offense propensity can justify the stepped-up implementation of institutionalization is a different matter. First of all, the higher rate can be a result of a defect in the mental health care system which cannot be considered the equivalent of the lack of institutionalization. This issue is especially important in Korea where institutionalization is dominant both in the number of beds and length of stay and involuntary admission has been abused by many people who were not eligible for admission. The higher rate of serious offense among persons with schizophrenia suggests the need for closer observation of this population, which would be achieved with close continuity of institutional and community care. Considering the factors which decrease patients’ compliance with treatment, such as side effects of medications, poor disease understanding, and reduced motivation for treatment, sustained observation with quality treatment and encouragement is required.

This study has several limitations to be considered. First, the mental status in the crime record in the KNPA statistics is based on the statements of the suspects and their families. Although this statement may be accompanied by the medical record and is examined by the police officer, it can be less accurate. Despite that, the comparison of the record with the statement concerning the disease in the court decision, which involved an exhaustive examination, showed that the figures presented in the crime record were reliable. Second, the number of persons with schizophrenia, which was calculated based on the records of health care utilization of the entire population in Korea, might have not captured the population which has not received the treatment while needing it. Third, although this study was intended to address the concern about crimes by people with schizophrenia, which were increased by a recent change toward deinstitutionalization, our study design, a cross-sectional comparison, has limitations in measuring the impact of the deinstitutionalization on crimes by people with schizophrenia. Lastly, the results in this study mainly concern the years before 2016 when the law concerning involuntary admission was revised. Therefore, the results should not be understood in connection with the loosened criteria for involuntary admission. Further studies should be performed to assess the impact of the latest revision. Our study should be understood as a cross-sectional description of the crimes committed by people with schizophrenia.

## Conclusions

This study examined the prevalence of crime in persons with schizophrenia in comparison with the general population. Persons with schizophrenia, despite their lower overall crime prevalence, showed a higher prevalence in murder, arson, and drug-related crimes. Whether it be a phenomenon stemming from intrinsic characteristics or caused by inappropriate care, it is evident that closer and more appropriate care, which would not leave the patients in danger of developing unfortunate consequences of their vulnerability, are required. This would be achieved through effective continuity of institutional and community care.

## Data Availability

The data we used are all publicly available. The KNPA crime statistics http://kosis.kr/statHtml/statHtml.do?orgId=132&tblId=DT_13204_4108&conn_path=I3 The Korean resident population http://kosis.kr/statHtml/statHtml.do?orgId=101&tblId=DT_1B040M5 The estimates of persons with schizophrenia in Korea http://www.hira.or.kr/download.do?src=%2Fshare%2Finternet%2Fpt%2Fbbs%2F479%2F2018%2F07%2FBZ201807233961031.pdf&fnm=2017+HIRA_11%EA%B6%8C+3%ED%98%B8_%EC%A1%B0%ED%98%84%EB%B3%91+%ED%99%98%EC%9E%90%EC%9D%98+%EC%B5%9C%EA%B7%BC+5%EB%85%84%EA%B0%84+%EC%A7%84%EB%A3%8C%EA%B2%BD%ED%96%A5+%EB%B6%84%EC%84%9D_%EB%B0%95%EA%B8%B0%EC%B0%AC.pdf

## Abbreviation

KNPA: Korean National Policy Agency
